# WHEN YOUR CAREGIVER IS ALSO YOUR CONFIDANT: CAREGIVER FUNCTIONS AND THE QUALITY OF CARE RECEIVED BY OLDER ADULTS

**DOI:** 10.1093/geroni/igae098.1291

**Published:** 2024-12-31

**Authors:** Yongxin Shang, Sarah Patterson

**Affiliations:** Cornell University, Ithaca, New York, United States; University of Michigan, Ann Arbor, Michigan, United States

## Abstract

Older adults can rely on multiple caregivers for support in later life and the characteristics of caregivers are closely related to the care received by older adults. This study aims to explore the association between caregiver functions and quality of care, asking if having a caregiver who also functions as a confidant can improve the care received by older adults. Using the baseline data from the National Health and Aging Trends Study (NHATS) in 2011, we construct an analytic sample of Medicare beneficiaries aged 65 or above with at least one caregiver to help with self-care (eating, bathing, toileting, dressing), mobility (going outside or inside, getting out of bed), or household activities (laundry, shopping, preparing meals, banking, medications) due to health or functioning reasons in the last month (N = 1,875). About 76% of these older adults identify at least one of their caregivers as their confidants, with whom they can discuss important things in the last year. OLS regressions on the care hours (log-transformed) suggest that older adults with at least one caregiver as a confidant are significantly more likely to receive more hours of care (p < 0.001). Logistic regressions on the binary outcomes of reporting any unmet care needs show that having a caregiver as a confidant is associated with a lower likelihood of having unmet care needs in household activities (p < 0.05), but not in self-care or mobility. These findings demonstrate the potential benefits of having a close tie as a caregiver in later life.

